# Palliative Care Program Development in a Low- to Middle-Income Country: Delivery of Care by a Nongovernmental Organization in India

**DOI:** 10.1200/JGO.17.00168

**Published:** 2018-03-13

**Authors:** Anjali Krishnan, M.R. Rajagopal, Safiya Karim, Richard Sullivan, Christopher M. Booth

**Affiliations:** **M.R. Rajagopal** and **Anjali Krishnan**, Trivandrum Institute of Palliative Sciences and Pallium India, Trivandrum, India; **Safiya Karim** and **Christopher M. Booth**, Queen’s University Cancer Research Institute, Kingston, Ontario, Canada; and **Richard Sullivan**, King’s College London and King’s Health Partners Comprehensive Cancer Centre, London, United Kingdom.

## Abstract

**Purpose:**

Limited data describe the delivery of palliative care services in low- and middle-income countries. We describe delivery of care by the Trivandrum Institute of Palliative Sciences (TIPS) in Trivandrum, India.

**Methods:**

Administrative records were used to describe case volumes, setting of care, and organizational expenditures. An estimate of cost per clinical encounter was derived by dividing 2016 monthly clinical expenditures by the number of patient visits. Costs are reported in US dollars and are corrected for Organization for Economic Co-operation and Development purchasing power parity (PPP).

**Results:**

A total of 11,620 new patients were seen at TIPS during 2007 to 2016; 59% had cancer. The average annual growth rate in case volumes was 18% (480 new patients in 2007 and 1,882 in 2016). The proportion of patients with cancer increased over time from 56% in 2014 to 66% in 2016 (*P* < .001). During 2014 to 2016, outpatient visits increased 26% (from 8,524 to 10,732), inpatient days increased 49% (from 1,763 to 2,625), inpatient visits at other hospitals increased 41% (from 248 to 417), and home visits increased 57% (from 3,951 to 6,186). Total clinical expenditures in 2016 were $288,489 (PPP corrected, $5.1 million). Between 2014 and 2016, the cost of delivering care increased by 74%. The mean cost per clinical encounter in 2016 was $15 (PPP corrected, $263).

**Conclusion:**

Demand for palliative care services has increased substantially, with an increasing proportion related to cancer. The organization of clinical services by TIPS may serve as a model for the development of other palliative care programs in low- and middle-income countries.

## INTRODUCTION

Poor access to effective palliative care is now recognized as a global issue with an urgent need for scaling up services, particularly in low- and middle-income countries (LMICs).^1-4^ An estimated 16 million people in LMICs each year will require end-of-life palliative care services. In addition to cancer, the demand for palliative care services is associated with the increasing burden of chronic and other noncommunicable diseases, such as HIV/AIDS, diabetes, and neurodegenerative diseases.^5^

The concept of palliative care is relatively new to India, after having been introduced in the 1980s. Since then, dedicated individuals, often in collaboration with international organizations, have developed hospice and palliative care services throughout the country. Throughout India, these services generally are located in large cities and associated with regional cancer centers. The south Indian state of Kerala has been a leader in the field of palliative care, with more widely available services provided by nongovernmental organizations (NGOs), public and private hospitals, and hospices since the 1980s.^4,6,7^

Kerala is home to the NGO Pallium India. This organization delivers clinical care, educates health professionals from across India, and serves as a national advocate for palliative care. Its flagship organization, the Trivandrum Institute of Palliative Sciences (TIPS), was founded in 2006 and offers inpatient, outpatient, and home-based community-oriented palliative care services. TIPS is a WHO collaborating center for training and policy on access to pain relief. Pallium India was instrumental in the adoption of the Palliative Care Policy by the Government of Kerala, making it the first LMIC government to have such a policy.^8^

Improved access to palliative care depends on the development of national health policies, a scale-up of existing services, and implementation of new health delivery systems.^4,9^ Rational design of health systems requires regional-level data and granular input from existing organizations currently delivering palliative care on the ground. There is a lack of published data to inform the design of palliative care delivery systems in the setting of the limited resources of most LMICs. To address this gap, this study has two objectives: to describe the population served and elements of clinical care delivered by TIPS and to provide an estimate of the economic costs of delivering this care. This information can inform the scaling up of palliative care services in other LMICs.

## METHODS

### Study Setting

TIPS was established in June 2006 with a mandate to deliver and demonstrate quality palliative care in Trivandrum, to provide palliative care education, and to engage in government advocacy for improved access to pain relief across India.^10^ The clinical services are delivered through a community-oriented palliative care model that includes an inpatient unit, outpatient clinics (on- and offsite), and home visits. TIPS works closely with other NGOs to form link centers in the community.

TIPS provides a 13-bed inpatient service at Arumana Hospital (AH). The inpatient service is organized into an acute ward where patients are admitted typically for 1 to 2 weeks for symptom management and/or end-of-life care. AH also serves as a halfway home where patients with spinal cord injuries are admitted for periods of 1 week to 3 months for rehabilitation. TIPS physicians also provide consultation services for inpatients in general wards at Medical College Hospital (MCH), a large public tertiary care hospital; Sri Avittam Tirunal Hospital (SAT), a public tertiary hospital for women and children; and General Hospital (GH), a large public hospital. Outpatient care is delivered in regular clinics at AH, MCH, GH, and SAT and in several community link centers located in peripheral villages. Outpatient clinics run 6 days a week in AH and MCH, 3 days a week in GH, 1 day a week in SAT, and 1 to 2 days a week in link centers. Overall, TIPS delivers care each week through 19 outpatient clinics and 18 home visit days attached to the community link centers. The home visits are conducted by four to five teams 5 days a week. Approximately half of the home visit teams are led by a physician and the other half by a nurse, along with an assistant who also serves as a driver.

A unique aspect of outpatient care in India is that a caregiver may attend the appointment instead of the patient to report on the patient’s condition and collect necessary medications. This is referred to as a proxy visit and is necessitated by difficult road conditions and inaccessibility for very ill patients. TIPS was founded with four staff members and now employs 57 individuals, including six palliative care physicians, 21 palliative care nurses, four medical social workers, three pharmacists, eight palliative care assistants (drivers and cleaning staff), and 15 administrative staff.

### Data Source

Basic demographic (age, sex) and clinical (cancer, noncancer diagnosis) information of each new patient seen by TIPS are entered into a Microsoft Excel (Microsoft Corporation, Redmond, CA) database. Daily visits of all patients (new and follow-up) are captured through case log sheets completed each day by the nurse attending home visits, the outpatient clinic, and the inpatient unit. In this study, we describe clinical case volumes from January 1, 2007 (the first full year of TIPS operations), through December 31, 2016. Description of the patient population, setting of care, and economic costs is restricted to the years 2014 to 2016 because these details were more readily available for the most recent years. Costs of delivering clinical care were identified from the Pallium India budget. Costs in rupees were converted to US dollars by using the exchange rate on December 31, 2016. These figures were corrected for purchasing power parity (PPP) by using the Organization for Economic and Co-operation and Development methodology.^11^ This study was approved by the institutional ethics committee of TIPS.

### Statistical Analysis

Differences in proportions over time were tested by using the Cochran-Armitage trend test. Results were considered statistically significant at *P* < .05. Analyses were performed by using Microsoft Excel and SAS version 9.3 (SAS Institute, Cary, NC).

## RESULTS

### Clinical Case Volumes and Patient Demographics

**Fig 1:**
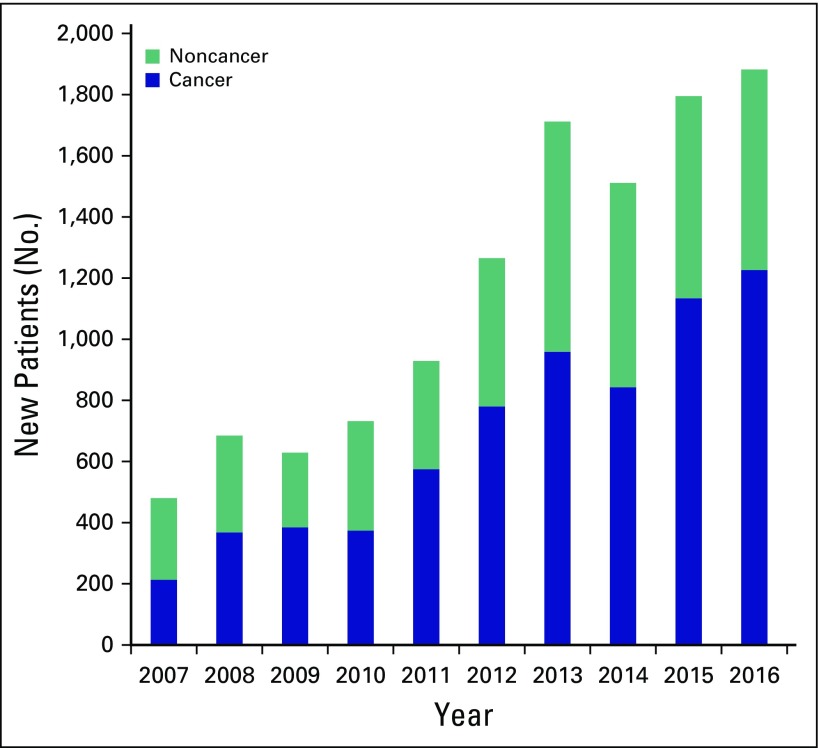
Annual case volumes for new patients treated by Trivandrum Institute of Palliative Sciences (2007 to 2016).

A total of 11,620 new patients were seen at TIPS during 2007 to 2016; 59% had cancer (n = 6,889) and 41% had noncancer (n = 4,809) diagnoses. During the past decade, there has been a fourfold increase (from 480 to 1,882 new patients/year) in the total number of new patients seen by TIPS (Fig 1). The volume of cancer-related diagnoses (six-fold increase from 213 to 1,226 patients/year) has increased at a greater rate than noncancer diagnoses (threefold increase from 267 to 6,565 patients/year). The average annual increase in case volume during 2007 to 2016 was 18%.

Demographic information was available for 5,188 new patients seen during 2014 to 2016 (Table 1). The median age was 63 years, and 56% were male. Seventy-six percent of patients were ages 41 to 80 years. New patients with cancer-related diagnoses accounted for 63% of the sample (n = 3,270) during 2014 to 2016; this proportion increased during each of the 3 years studied (from 56% to 63% to 66%; *P* < .001). Noncancer diagnoses accounted for 37% of new patients (n = 1,985) seen during 2014 to 2016. The most common noncancer diagnoses were frailty (20% [n = 389]), stroke (17% [n = 330]), heart disease (13% [n = 254]), and neurologic disorders (8% [n = 155]; Table 2).

**Table 1:**
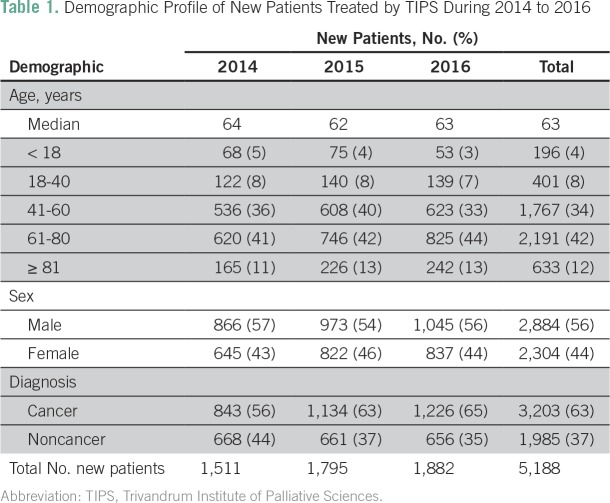
Demographic Profile of New Patients Treated by TIPS During 2014 to 2016

**Table 2:**
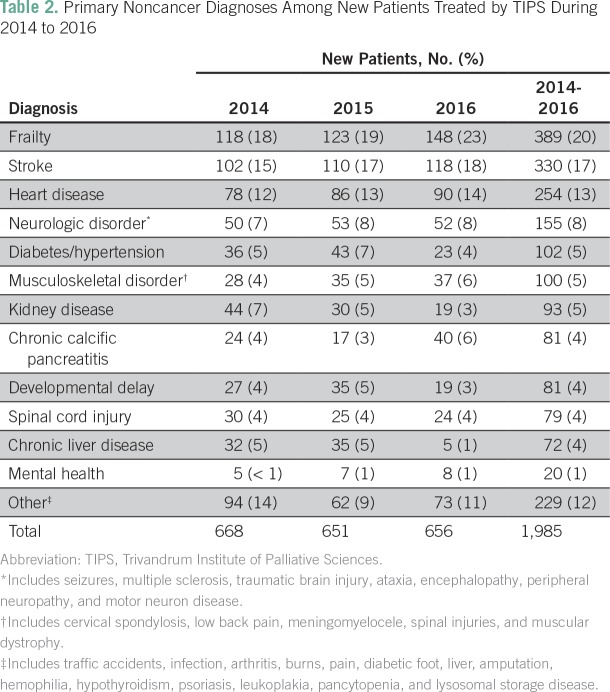
Primary Noncancer Diagnoses Among New Patients Treated by TIPS During 2014 to 2016

### Care Delivery Setting

**Fig 2:**
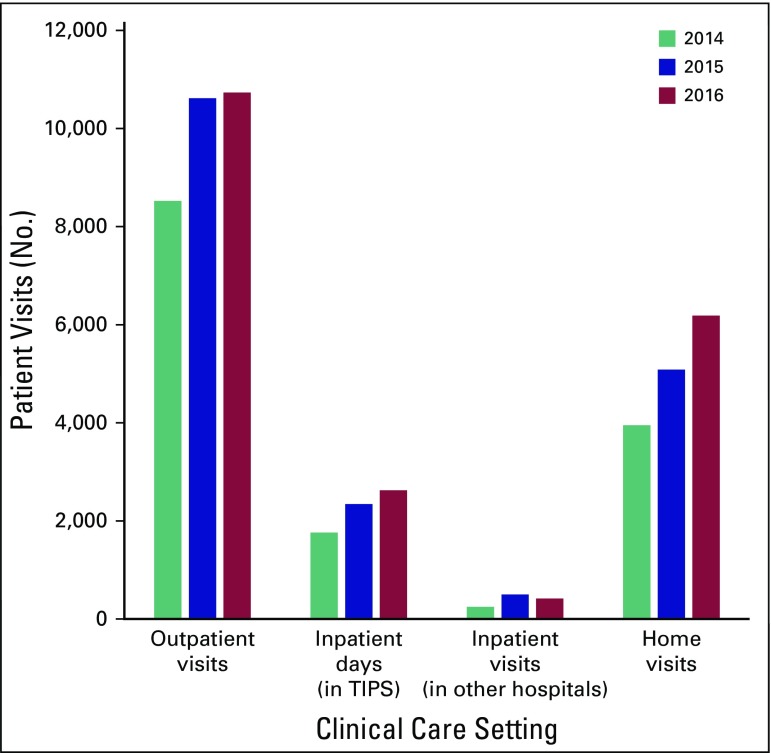
Setting of clinical care delivered by Trivandrum Institute of Palliative Sciences (TIPS) during 2014 to 2016.

As shown in Figure 2 and Table 3, the majority of clinical care is delivered in the outpatient clinics and through home visits (56% and 29% of clinical encounters, respectively). During 2014 to 2016, there were 29,874 outpatient visits, of which 37% involved a patient proxy (n = 10,963). Fifty percent of outpatient visits (n = 14,811) were conducted in the community link centers; 31% (n = 9,133) were carried out in the government MCH, 13% (n = 3,888) in AH (headquarters of TIPS), 6% (n = 1,682) in the women/child government hospital SAT, and 1% (n = 360) in the government GH. Outpatient case volumes increased substantially from 8,524 visits in 2014 to 10,732 visits in 2016 (26% increase; *P* < .001). A total of 15,221 home visits took place during 2014 to 2016; case volumes increased substantially by 57% from 3,951 in 2014 to 6,186 in 2016.

**Table 3:**
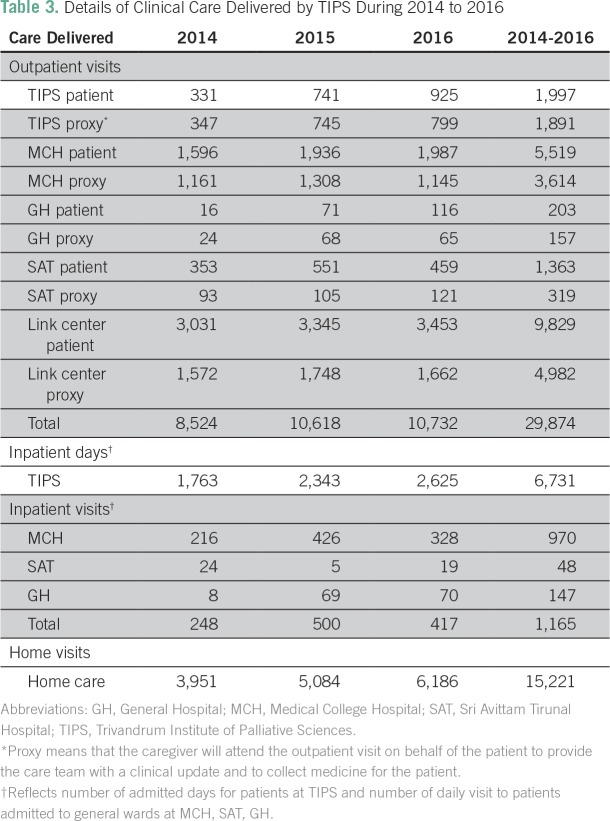
Details of Clinical Care Delivered by TIPS During 2014 to 2016

During 2014 to 2016, TIPS provided care to inpatients at AH for a total of 6,731 patient-days. In addition, 1,165 inpatients were seen in consultation at MCH, SAT, and GH. During this 3-year period, inpatient care at AH increased by 49% (1,763 to 2,625 patient-days); inpatient visits in general wards at other hospitals increased by 40% (from 248 to 417 patients).

### Delivery of Nonclinical Support

TIPS provides a variety of additional support to patients, which includes vocational rehabilitation training (ie, catering technology course, computer skills, soap manufacturing), educational support for children of patients’ families who are at risk for dropping out of school, and food kits for families at risk for starvation. During 2007 to 2016, TIPS provided financial support for approximately 1,598 years of schooling. After secondary school, 79 students have been supported through professional degree programs, including nursing, medicine, engineering, journalism, and business management. Currently, 307 children are receiving educational support (281 up to secondary school and 26 for professional courses), and 54 families are receiving monthly food kits.

### Costs of Delivering Clinical Care

**Fig 3:**
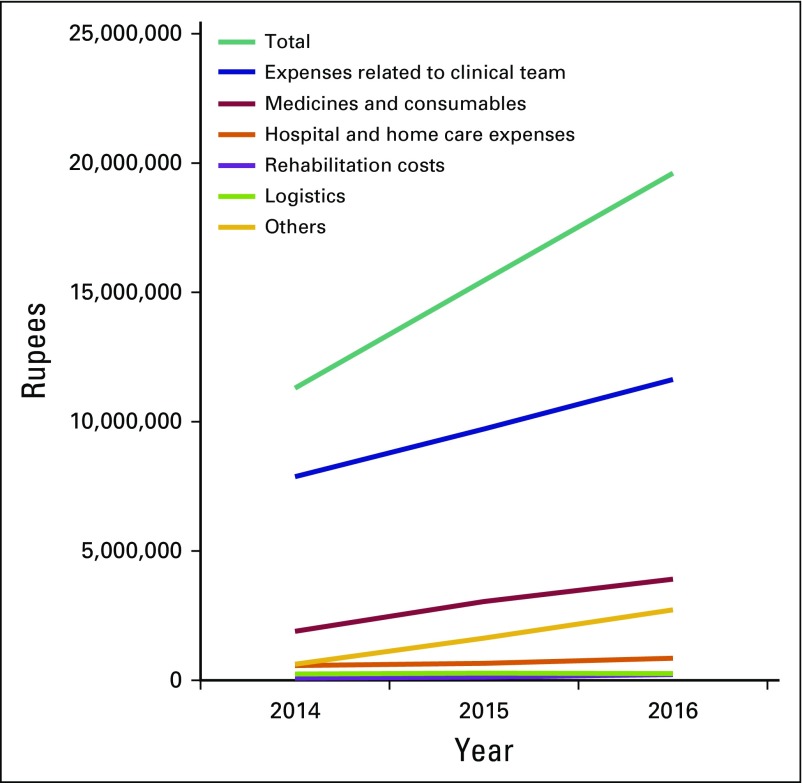
Annual clinical expenditures of Trivandrum Institute of Palliative Sciences (2014 to 2016). Expenses related to clinical team are salary, benefits, and nursing uniforms. Rehabilitation costs are wheelchairs, crutches, vocational rehabilitation, and food kits. Logistics include repairs and maintenance of vehicles and insurance. Other expenses are Arumana Hospital rent and utilities.

Total expenditure by TIPS in 2016 was 19.6 million rupees ($288,773); the PPP correction is $5.1 million (Data Supplement). With 1,663 clinical encounters per month, this translates to $15 per visit (PPP correction, $263 per visit). Sixty percent of total expenses are related to human resources (clinical personnel, including physicians, nurses, social workers, and palliative care assistants), and 20% is spent on medicines and other consumables (ie, catheters, bed pans, blood pressure cuffs, sterile and nonsterile gloves, dressing materials). The remaining 20% of the expenses are attributable to infrastructural hospital costs, rehabilitation supplies (ie, wheelchairs, crutches, vocational rehabilitation, food kits), repair and maintenance of vehicles, and rent and utilities. During 2014 to 2016, total expenses increased by 72% (from 11.4 million to 19.6 million rupees). This increase has been driven largely by increased spending on medicines and consumables (106% increase) and an increased number of staff salaries (48% increase; Fig 3).

## DISCUSSION

We describe palliative care services delivered by TIPS in the Indian province of Kerala. Several important findings emerged. In the past decade, there was substantial growth in case volumes largely driven by an increase in cancer-related diagnoses. These trends are a worldwide phenomenon.^12^ Over the past 3 years, outpatient volumes have grown by > 30%. A substantial number of outpatient visits are attended by patient proxy. Inpatient care at AH also increased over time, as has the provision of nonclinical support, such as vocational training, educational stipends, and food kits. This study also presents data from a rudimentary costing exercise to underscore the investment required to deliver these services. The model of care for palliative services described here represents affordable and high-value care; these data provide an economic basis upon which states and union ministers can plan the expansion of such services.

The growth in case volume is not surprising. Any palliative care program in an underserved area will have similar exponential growth with increasing awareness of service availability. The second author (M.R.R.) has had similar experience with a fledgling palliative care service in the 1990s.^13^ Although palliative care is more advanced in Kerala than in other parts of India, coverage is still poor as evidenced by per capita morphine consumption, which is an indicator for access to palliative care. In 2013, the per capita morphine consumption in Kerala was 1.1 mg. Although morphine consumption was 10-fold higher than the national average in India (0.11 mg per capita), it was only one sixth of the global average (6.3 mg) and only 0.5% of that in the United Kingdom (241 mg per capita).^14,15^ These data suggest that existing palliative care services in India are meeting only a small minority of the clinical need. To address the massive shortfall in access will require broad structural reform of palliative care across India by using programs like TIPS as models and integrating palliative care as core to existing and expanding secondary and tertiary health care centers in the country.

Data from the current analysis identified a cost per clinical encounter of $15 (PPP, $263). Despite the difficulty in determining an appropriate comparison with other palliative care programs, this cost compares favorably with other reports. In a literature review of palliative care costs from high-income countries, average daily variable costs ranged from $130 to $897 for hospital and home-based palliative care programs; total palliative care costs per year ranged from $11,741 to $14,486 per patient.^16^

The current study results should be considered in light of methodological limitations. Details of symptom burden, treatment delivered, and TIPS referral patterns are not available from existing administrative databases. Our costing exercise did not distinguish the inherent differences in costs across outpatient versus inpatient settings. This analysis was purposefully rudimentary because of a lack of detailed economic data. The objective was to illustrate overall costs at a high level to facilitate discussions among other providers and payers in LMICs. Finally, the increase in cancer versus noncancer diagnoses is based on the 3 years for which we have detailed clinical data (2014 to 2016) and, therefore, may not represent long-term trends. However, with economic development and epidemiologic transition, the cancer burden in many LMICs will increase; planning for future palliative care services in LMICs will need to consider this shift in disease prevalence.

Funding for TIPS comes from a variety of sources but primarily from philanthropic donations; a smaller portion of funding comes from project grants. Unsolicited donations from families of patients who have been treated by TIPS is one of the major sources of support. Some business houses regularly contribute to Pallium India’s work. The organization’s fleet of eight vehicles used for home visits were provided by national banks (as part of corporate social responsibility efforts) and philanthropic organizations. Since 2015, one specific activity—the halfway home for people with spinal injury—has been supported by the Department of Social Justice, Government of Kerala.

The sex case mix (56% male, 44% female) in our cohort raises concerns about equality in access to care; 51% of Kerala’s population is female,^17^ which may reflect the broader issue of gender empowerment in India and equality in health service access.^18^ Kerala generally is considered to be among the most progressive states in India, which suggests that this observation might be even more striking elsewhere in the country. The sociocultural environment of many LMICs is such that although palliative care is sought for one individual, delivery of holistic care requires consideration of the entire family unit. When one parent dies, it is not uncommon for the remaining family to be thrust into abject poverty, which often leads to a cascade of despair as children drop out of school in an effort to gain income for the remaining family members. To address this problem, TIPS provides ongoing support for education of children in the patient’s family that may be provided for years after the patient’s death.

In India, up to 75% of health care costs come out of pocket^18^; therefore, it is common for disease-specific treatment to lead to a vicious cycle of financial ruin and poverty. Out-of-pocket expenditures remain a major source of catastrophic expenditures in India^18^ that are largely driven by a medical system that emphasizes a search for futile curative treatment. For this reason, one of the policy aims of TIPS is to educate patients, families, and the public about the benefits of high-quality end-of-life care, which may reduce the risk of financial ruin so common in this setting.

One of the greatest successes of TIPS has been the engagement of community and support of volunteers. A network of volunteers supports TIPS in a variety of roles, including registering a local NGO to ensure sustainability, finding a local venue to house the outpatient facility, assisting with nursing chores, acting as a link between the patient and family, and providing psychosocial support. Beyond the immediate comfort these volunteers provide to patients and families, they have been instrumental in contributing to the growing profile of the palliative care movement. This grassroots movement culminated in state government acceptance of a proposal by Pallium India that led to the Kerala’s Pain and Palliative Care Policy in 2008.^19^ To our knowledge, Kerala is the first LMIC government to have a dedicated policy on palliative care.

A major weakness of TIPS is that it is a stand-alone facility and thus offers a poor opportunity for integration of palliative care into the health care system. Although TIPS delivers outpatient palliative care services in government hospitals, it has made only slow inroads into hospital practices. Public awareness is steadily improving about palliative care, but the general perception is that it is meant only for the care of the dying, an unfortunate factor that limits access to symptom control and psychosocial spiritual support early in the course of disease.

Given the vast need for palliative care in the community, a balance of quality care and coverage is challenging. Pallium India’s initiative, which resulted in the creation of a minimum standards tool in palliative care, is aimed at drawing a line below which the service should not drop. Nevertheless, the line seems too low, and services often are inadequate. A weekly home visit is hardly sufficient to ensure reasonable quality of care for a patient with advanced cancer. Currently, this deficiency is partially mitigated by providing access to a 24/7 inpatient facility to cater to pressing needs. However, much work remains to increase access and quality of care. Although the frequency and duration of clinical visits will vary on the basis of patient need, most patients are seen approximately twice a month, and clinic/home visits usually are 30 to 45 minutes long. Management is driven by individual symptom assessments. The most common clinical problems are pain, dyspnea, nausea/vomiting, constipation/bowel obstruction, delirium, and fungating wounds. Immediate-release morphine is used for moderate to severe pain (step 3 on the WHO cancer pain ladder). Neuropathic pain is commonly managed with tricyclic antidepressants and anticonvulsants such as sodium valproate, which are cheaper than gabapentin and pregabalin. Common procedures offered by the TIPS team include pressure sore/malignant wound management, manual rectal evacuation/enema, thoracentesis/paracentesis, subcutaneous and intravenous fluid administration, bladder catheterization, nasogastric tube insertion, ostomy care, and lymphedema care.

Data from the current study and our previous work in morphine use^14^ illustrate the urgent need to expand palliative care services within the government sector. Development of health systems that improve access to palliative care (especially in district medical college hospitals and community health centers) will improve quality of life for patients and mitigate the cascade of suffering and poverty that is so common among remaining family members. Policymakers must address palliative care as an essential entity and take necessary steps to improve access through establishing palliative care units in secondary and tertiary health centers and to develop a health insurance scheme to support the delivery of high-quality palliative care.
